# LncRNA-Mediated miR-145 Sponging Drives FN1 and CCND1 Expression: Prognostic and Therapeutic Targets in NSCLC

**DOI:** 10.3390/biom15111564

**Published:** 2025-11-06

**Authors:** Safa Tahmasebi, Davar Amani, Babak Salimi, Ian M. Adcock, Esmaeil Mortaz

**Affiliations:** 1 Department of Immunology, School of Medicine, Shahid Beheshti University of Medical Sciences, Tehran 19839-69411, Iran; safa.tahmasebi@sbmu.ac.ir; 2 Research Center of Thoracic Oncology (RCTO), National Research Institute of Tuberculosis and Lung Diseases (NRITLD), Shahid Beheshti University of Medical Sciences, Tehran 19839-69411, Iran; babak.salimi@sbmu.ac.ir; 3 Respiratory Section, Faculty of Medicine, National Heart and Lung Institute, Imperial College London, London SW7 2AZ, UK; adcock.IM@imperial.ac.uk; 4 Respiratory Immunology Research Center, National Research Institute of Tuberculosis and Lung Diseases (NRITLD), Shahid Beheshti University of Medical Sciences, Tehran 19839-69411, Iran

**Keywords:** non-small cell lung cancer, ceRNA network, long non-coding RNA, miR-145, fibronectin 1, CCND1

## Abstract

**Background:** Non-small cell lung cancer (NSCLC) progression is driven by dysregulated competing endogenous RNA (ceRNA) networks, where non-coding RNAs sequester miRNAs to modulate oncogene expression. The tumor-suppressor miR-145 is frequently downregulated in NSCLC, but its lncRNA-mediated regulation remains incompletely characterized. **Methods:** Integrated transcriptomic analysis of NSCLC datasets (GSE135304: blood RNA from 712 patients; GSE203510: plasma miRNAs) was used to identify dysregulated genes (|log2FC| > 0.1, *p* < 0.05) and miRNAs (|log2FC| > 1, *p* < 0.05). Experimentally validated targets from miRTarBase/TarBase were intersected with dysregulated genes, followed by WikiPathways/GO enrichment. ceRNA networks were constructed via co-expression analysis. RT-qPCR validated miR-145-3p expression in A549/MRC-5 cells and NSCLC tissues. GEPIA assessed FN1/CCND1 clinical relevance. **Results:** We identified 8271 dysregulated genes and 52 miRNAs. miR-145-3p, critical in immune regulation, was significantly downregulated (log2FC = −1.24, *p* = 0.036). Intersection analysis revealed 27 miR-145-3p targets (e.g., FN1, CCND1, SMAD3) enriched in immune pathways (FDR < 0.05) and TGF-β-mediated EMT within the dysregulated geneset. Six immune-linked hub genes emerged. LncRNAs LOC729919 and LOC100134412 showed strong co-expression with hub genes and competitively bind miR-145-3p, derepressing the expression of the metastasis drivers FN1 (ECM regulator) and CCND1 (cell cycle controller). This ceRNA axis operates within a broader dysregulation of ATM-dependent DNA damage, Hippo signaling, and cell cycle pathways. RT-qPCR confirmed significant miR-145-3p suppression in NSCLC models (*p* < 0.05). GEPIA revealed a significant FN1-CCND1 co-expression (*p* = 0.0017). **Conclusions:** We characterize a novel LOC729919/LOC100134412–miR-145–FN1/CCND1 ceRNA axis in NSCLC pathogenesis. FN1’s prognostic value and functional linkage to CCND1 underscores its potential clinical relevance for therapeutic disruption.

## 1. Introduction

Non-small cell lung cancer (NSCLC) represents approximately 85% of all lung malignancies and persists as the leading cause of cancer-related mortality globally, with an estimated 1.8 million deaths annually [1,2]. Prognosis remains extremely poor for advanced-stage disease, where 5-year survival rates are <10% despite therapeutic advances [3]. Clinical outcomes are dictated by a confluence of factors, including stage at diagnosis, molecular driver alterations (e.g., EGFR, ALK, ROS1), and histological subtype [3]. Smoking history, performance status, and emerging liquid biopsy biomarkers such as circulating tumor DNA further refine risk stratification, but underscore the urgent need for novel mechanistic insights into NSCLC progression [4].

The competing endogenous RNA (ceRNA) regulatory paradigm has emerged as a critical layer of post-transcriptional control in NSCLC pathogenesis [5,6]. This network hinges on competitive miRNA sequestration by lncRNAs, pseudogenes, or circular RNAs, which subsequently derepress oncogenic mRNA targets [7]. Seminal studies have established clinically relevant ceRNA axes: for example, the lncRNA MALAT1 drives metastasis by sponging miR-204 to elevate SLUG expression, while HOTAIR promotes invasion through miR-326-mediated TCF7 upregulation [8,9]. Notably, such networks frequently modulate therapeutic resistance; LINC01559 confers osimertinib resistance by adsorbing miR-320a to stabilize IGF2BP3, and UCA1 enhances platinum resistance via miR-193a-3p/ERBB4 signaling [10,11]. These findings position ceRNA dysregulation as a hallmark of NSCLC malignancy and drug response.

Despite growing recognition of ceRNA networks in NSCLC pathogenesis, critical voids persist in their functional characterization. Over 70% of dysregulated transcripts in NSCLC remain unannotated, and their roles as lncRNA sponges, particularly in regulating metastasis-driving extracellular matrix (ECM) effectors such as fibronectin (FN1), are largely unexplored. This knowledge gap impedes the discovery of therapeutic targets for advanced disease. Our study therefore employs integrated transcriptomics of malignant nodules to systematically reconstruct the NSCLC ceRNA interactome, prioritizing uncharacterized lncRNAs and their roles in ECM dysregulation, with the goal of uncovering novel targetable vulnerabilities.

## 2. Methods

### 2.1. Data Acquisition and Pre-Processing

Two transcriptomic datasets were retrieved from the Gene Expression Omnibus (GEO) database to investigate immune-related ceRNA networks in non-small cell lung cancer (NSCLC). The GSE135304 dataset comprised blood RNA profiles from 712 patients (404 with malignant pulmonary nodules; 220 with benign nodus; 88 with no nodules), while GSE203510 contained plasma miRNA profiles from 20 subjects (10 NSCLC patients; 10 healthy donors). Both datasets underwent quantile normalization using the normalizeQuantiles function in R (v4.2.1) to minimize technical variation, with normalization efficacy confirmed through box plot visualization.

### 2.2. Identification of Differentially Expressed Genes and miRNAs

Differential expression analysis was performed using the limma package. For gene expression data (GSE135304), thresholds of |log_2_FC| > 0.1 and *p* < 0.05 were applied. For miRNA profiling (GSE203510), stringent thresholds of |log_2_FC| > 1 and *p* < 0.05 were used.

### 2.3. Target Prediction and Intersection Analysis

hsa-miR-145-3p was prioritized based on immune system relevance. Experimentally validated mRNA targets of hsa-miR-145-3p were predicted using the multiMiR package (v1.18.0), integrating interactions from miRTarBase (v9.0) and TarBase (v8.0).

### 2.4. Functional Enrichment Analysis

The intersection between DEGs from GSE135304 and predicted targets from multiMiR was determined. Pathway enrichment analysis of overlapping genes was performed using the Enrichr database with WikiPathways. Gene Ontology (Biological Process, Cellular Component, Molecular Function) terms were analyzed.

### 2.5. ceRNA Network Construction

The Hmisc R package (v4.7) was used to calculate Pearson correlation coefficients between immune-related genes and lncRNAs in the GSE135304 expression matrix. LncRNAs with absolute correlation coefficients > 0.5 were selected for ceRNA network construction. Networks were visualized using Cytoscape (v3.9.1).

### 2.6. RT-qPCR Validation of miR-145 in NSCLC Tissues and Cell Lines

The present study obtained the Institutional Ethical Committee and Research Advisory Committee of Shahid Beheshti University of Medical Sciences (IR.SBMU.MSP.REC.1402.397). The obtained date of approval code was 24 October 2023. Also, to take the patients’ tissues, informed written consent has been obtained for participation in the study. Total RNA was isolated from paired tumor and normal lung tissues of seven NSCLC patients (collected during curative resection) and cultured cell lines using RNX Plus solution (SinaClone, Tehran, Iran; Cat# EX6101) according to the manufacturer’s protocol. The NSCLC cell line A549 [(ATCC^®^ CCL-185™) and (RRID: CVCL_0023)] and normal lung cell line MRC-5 [(ATCC^®^ CCL-171™) and (RRID: CVCL_ 0440)] were purchased from the Pasteur Institute of Iran. Both human cell lines were tested for lung carcinoma and characterized by STR-profiling as indicated by the DSMZ online catalogue [12]. Cell lines were maintained in DMEM supplemented with 10% fetal bovine serum at 37 °C/5% CO_2_. RNA concentration and purity was verified using NanoDrop 2000c (Thermo Scientific, Waltham, MA, USA; A260/A280 ratio 1.8–2.0) with subsequent storage at −80 °C.

For miRNA analysis, cDNA was synthesized from 1 μg total RNA using a poly(A)-tailing-based miRNA cDNA synthesis kit (Bon Yakhteh, Tehran, Iran). Quantitative real-time PCR was performed using SYBR Green Master Mix (Ampliqon, Odense, Denmark) on Applied Biosystems StepOne Real-Time PCR Systems with the following cycling conditions: 95 °C for 15 min (initial denaturation), followed by 40 cycles of 94 °C for 15 s, 55 °C for 30 s, and 70 °C for 30 s. Stem-loop primers specific for hsa-miR-145-3p (Bon Yakhteh, Tehran, Iran) were employed for amplification. The small nuclear RNA U48 (RNU48) served as the endogenous control. Relative expression was calculated using the 2^−ΔΔCt^ method. All experiments were performed in triplicate with statistical significance determined by Student’s *t*-test (*p* < 0.05) in GraphPad Prism v9.0.

### 2.7. GEPIA Survival and Correlation Analysis

To validate the clinical relevance of FN1 and CCND1 within the identified ceRNA axis, we performed secondary analysis using the Gene Expression Profiling Interactive Analysis (GEPIA) platform. This public resource integrates RNA-seq data from 9736 tumors and 8587 normal samples across 33 cancer types from The Cancer Genome Atlas (TCGA) and Genotype-Tissue Expression (GTEx) projects. For non-small cell lung adenocarcinoma (LUAD), we conducted survival analysis using Kaplan–Meier curves with log-rank testing to compare overall survival between high (top 50%) and low (bottom 50%) expressers of FN1 and CCND1. Additionally, Pearson correlation analysis was performed to examine co-expression patterns between FN1 and CCND1 mRNA levels across NSCLC tumors. In addition, progression-free interval (PFI) analysis for FN1 and CCND1 was performed using the GEPIA3 online tool “https://gepia3.bioinfoliu.com/ (accessed on 9 September 2025)”, which employs a multivariate Cox proportional hazards model. Statistical significance was defined at *p* < 0.05, with hazard ratios and correlation coefficients reported.

### 2.8. Statistical Analysis

All statistical analyses were implemented in R (v4.2.1). Differential expression significance was defined as *p* < 0.05, with correlation significance determined at the same threshold.

## 3. Results

### 3.1. Demographic and Clinical Characteristics of the Study Cohort

The GSE135304 cohort comprised 712 non-small cell lung cancer patients, including 382 females and 330 males, with a mean age of 65.3 years and a broad age range spanning from 26 to 93 years. Smoking history analysis revealed that 29 patients had never smoked, 461 were former smokers, and 219 were active smokers. Tumor staging distribution showed 234 cases at Stage I, 44 at Stage II, 60 at Stage III, 46 at Stage IV, and 108 cases with unspecified stage.

**Figure 1:**
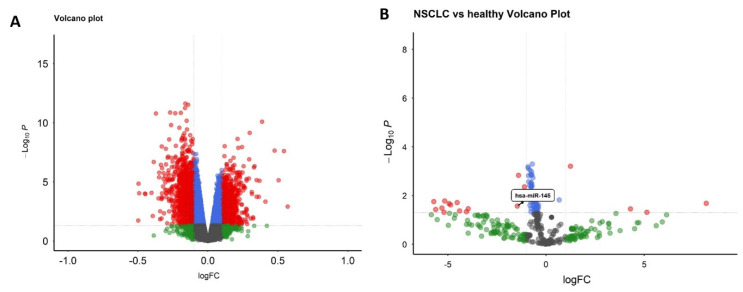
Differential expression in NSCLC: (**A**) genes (GSE135304), (**B**) miRNAs (GSE203510).

Differential expression analysis identified 8271 significantly dysregulated genes between malignant nodule and non-nodule patients, visualized through a volcano plot highlighting prominent transcriptional alterations (Figure 1A). miRNA profiling demonstrated 52 differentially expressed miRNAs distinguishing NSCLC patients from healthy controls in the GSE203510 dataset (Figure 1B).

### 3.2. Functional Enrichment of Differentially Expressed Genes

Pathway enrichment analysis of differentially expressed genes using WikiPathways identified the ATM-dependent DNA damage response as the most significantly dysregulated pathway, followed by TGF-β signaling, cell cycle regulation, Hippo signaling, and TGF-β-mediated epithelial–mesenchymal transition pathways. Key genes recurrently appearing across multiple pathways included FN1 and SMAD3, suggesting their central role in malignant progression (Table 1).

### 3.3. Selection of hsa-miR-145-3p and Target Identification

hsa-miR-145-3p was prioritized as the central miRNA regulator based on literature-established immune system relevance. This miRNA showed significant downregulation with log2 fold change of −1.24 and *p*-value of 0.036. Integration of experimentally validated miR-145 targets from miRTarBase and TarBase yielded 121 target mRNAs, which when intersected with differentially expressed genes from the malignant nodule cohort, identified 27 overlapping targets: POTED, GK5, CDC23, SFT2D2, SMAD3, ANGPTL3, SERPINA1, ESRRG, ZFP36L2, BUB1, CDK5R1, PRKAG1, ARHGAP21, HECTD2, FN1, ZC3H6, TBC1D8, CCND1, RAB11FIP1, PURB, CKAP4, TRIM23, SIGLEC14, ZNF281, OGT, VCAM1, and CCND2.

### 3.4. Pathway and Functional Enrichment

**Figure 2:**
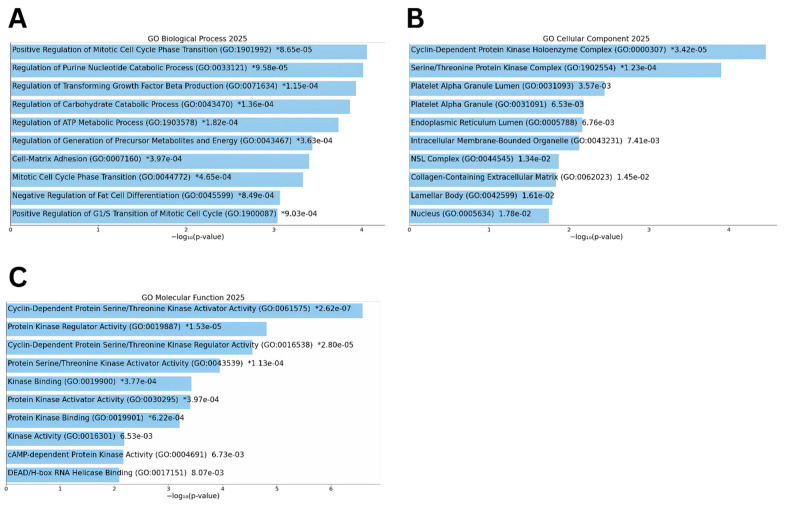
Enrichment of 27 miR-145-3p targets: (**A**) Biological Process (BP), (**B**) Cellular Component (CC), (**C**) Molecular Function (MF) GO terms.

Pathway analysis of the 27 overlapping genes identified significant enrichment in immune-related pathways using WikiPathways. Gene Ontology results appear in Figure 2 with biological process terms shown in panel A, cellular component terms in panel B, and molecular function terms in panel C (Table 2).

### 3.5. Immune-Related Network Visualization

**Figure 3:**
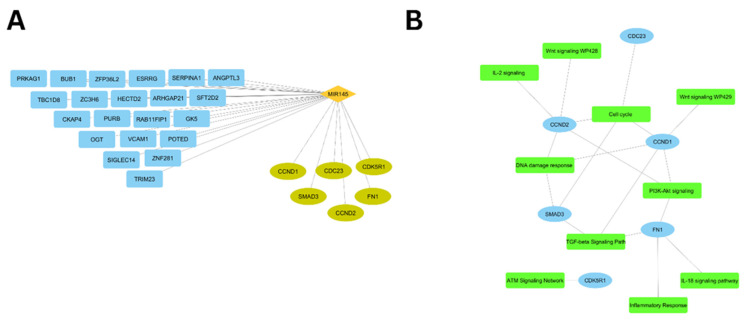
(**A**) miR-145-3p interaction network with 27 target genes: immune-related genes (turquoise, lower right) and other targets (blue). (**B**) Pathway-focused network of six immune-related hub genes.

Two distinct networks elucidate the regulatory relationships. Figure 3A presents the network of miR-145-3p interactions with all 27 target genes, where immune-related genes appear in turquoise positioned at the lower right quadrant and remaining targets in blue. The Hmisc R package was applied to the filtered gene expression matrix from GSE135304 to calculate Pearson correlation coefficients between the 6 immune-related hub genes and lncRNAs. Figure 3B displays the pathway-centric network of the 6 immune-related genes.

### 3.6. ceRNA Network Construction

**Figure 4:**
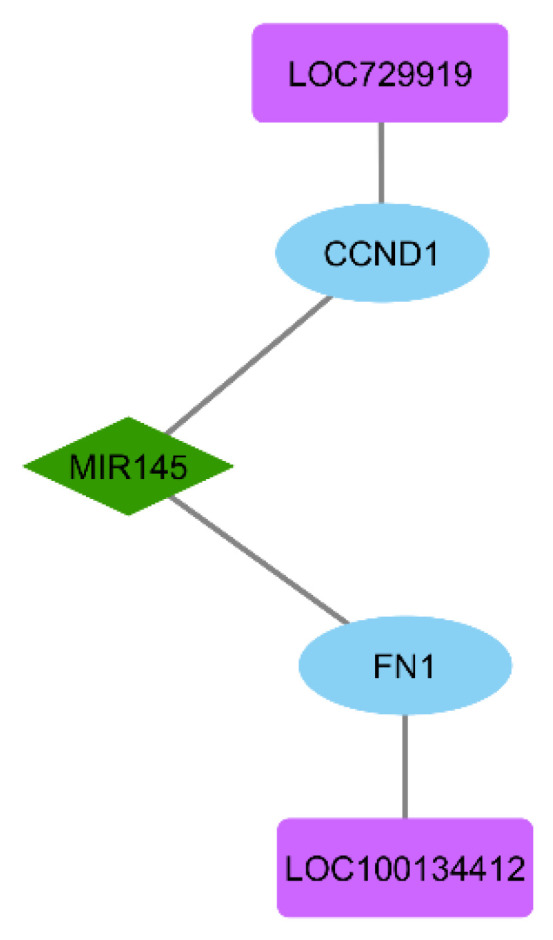
ceRNA axis: LOC729919/LOC100134412 sponge miR-145-3p to derepress FN1 and CCND1.

LncRNAs demonstrating absolute correlation coefficients exceeding 0.5 were selected for ceRNA network construction. This analysis identified LOC729919 and LOC100134412 as the top regulatory lncRNAs with significant co-expression relationships. The final ceRNA network (Figure 4) integrates the lncRNA sponges LOC729919 and LOC100134412 with miR-145-3p and their key targets (FN1 and CCND1).

### 3.7. miR-145 Downregulation in NSCLC Tissues and Cell Lines via RT-qPCR

**Figure 5:**
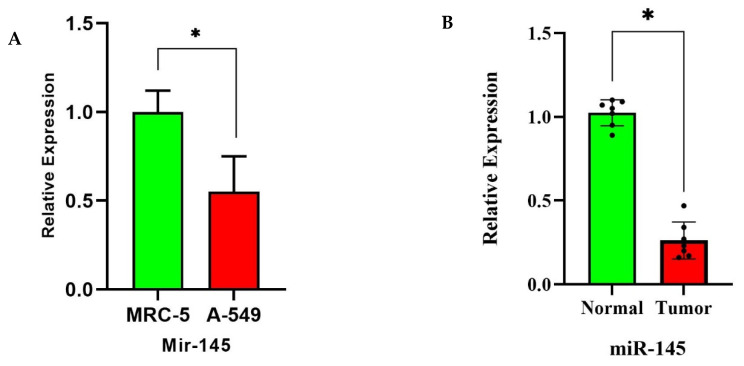
miR-145-3p downregulation in NSCLC: (**A**) A549 vs. MRC-5 cell lines (*: *p* < 0.028); (**B**) tumor vs. normal tissues (*: *p* < 0.037).

RT-qPCR analysis confirmed significant suppression of miR-145-3p across NSCLC models. In cell line comparisons, A549 (NSCLC) cells exhibited a significant reduction in miR-145-3p expression relative to MRC-5 normal lung cell line (*p* < 0.028) (Figure 5A). Similarly, tumor tissues from NSCLC patients demonstrated a significant decrease compared to matched normal lung tissues (*p* < 0.037) (Figure 5B).

### 3.8. Clinical Validation of FN1/CCND1 Relevance via GEPIA

**Figure 6:**
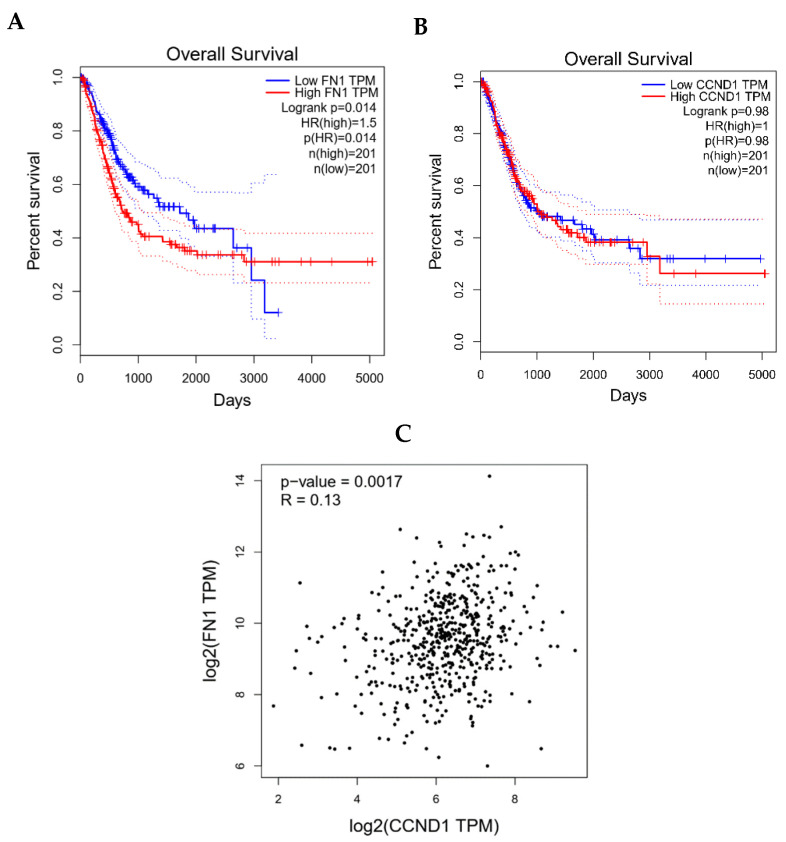
Clinical relevance of FN1 and CCND1 in NSCLC via GEPIA: (**A**) adverse survival with high FN1 expression, (**B**) no survival association for CCND1, (**C**) significant FN1-CCND1 co-expression.

GEPIA revealed distinct clinical implications for FN1 and CCND1 in lung adenocarcinoma. High FN1 expression significantly correlated with poorer overall survival, exhibiting a hazard ratio of 1.5 and log-rank *p*-value of 0.014, indicating its potential as an adverse prognostic biomarker (Figure 6A). In contrast, CCND1 expression levels showed no association with patient survival outcomes (Figure 6B). Notably, a statistically significant positive correlation was observed between FN1 and CCND1 mRNA expression across NSCLC tumors, with a Pearson correlation coefficient of 0.13 and *p*-value of 0.0017 (Figure 6C). Moreover, progression-free interval (PFI) analysis revealed that high expression of FN1 was significantly associated with poorer PFI in NSCLC patients (HR = 1.077, *p* = 0.028), while CCND1 expression showed no significant correlation with PFS (HR = 1.022, *p* = 0.639).

## 4. Discussion

NSCLC represents approximately 85% of all lung cancer cases and remains the leading cause of cancer-related mortality worldwide, with a 5-year survival rate below 20% for advanced stages [13,14]. The disease manifests as adenocarcinoma (40%), squamous cell carcinoma (25–30%), and large cell carcinoma (5–10%), each with distinct pathological features and molecular drivers [15]. Major risk factors include cigarette smoking—responsible for 80% of cases—alongside genetic predispositions (e.g., TP53 germline variants), radon exposure, and air pollution [3,16]. Despite advancements in targeted therapies and immunotherapy, most patients present with locally advanced or metastatic disease, underscoring the need for novel diagnostic biomarkers and therapeutic targets. Recently, the competing endogenous RNA (ceRNA) hypothesis reveals how RNA species—particularly long non-coding RNAs (lncRNAs)—orchestrate gene expression by sequestering microRNAs (miRNAs), thereby modulating messenger RNA (mRNA) targets [5]. These regulatory networks are increasingly recognized as central players in cancer pathogenesis, influencing oncogenic pathways like epithelial–mesenchymal transition (EMT) and immune evasion through dynamic RNA crosstalk [17,18].

Our integrated transcriptomic and experimental analyses reveal a novel ceRNA regulatory axis in NSCLC pathogenesis, wherein the lncRNAs LOC729919 and LOC100134412 function as molecular sponges for miR-145-3p, thereby derepressing the expression of the metastasis-associated ECM regulator FN1 and the cell cycle controller CCND1. We demonstrate significant downregulation of miR-145-3p in both NSCLC cell lines and patient tissues, reinforcing its tumor-suppressive role. Critically, clinical database analysis establishes FN1 as a prognostic biomarker, with elevated expression correlating with adverse survival outcomes, and reveals significant co-expression between FN1 and CCND1 across NSCLC tumors, supporting their functional synergy within this network.

Our integrated bioinformatics analysis identified miR-145 as significantly downregulated in NSCLC tissues and cell lines compared to normal controls. Further, our experimental validation confirmed a marked downregulation of this miRNA in both cellular and clinical NSCLC models, reinforcing its well-established role as a tumor suppressor across multiple malignancies [19,20,21]. This suppression is mechanistically linked to NSCLC metastasis through miR-145’s targeting of key oncogenes involved in invasion (MMP9, FSCN1), stemness (OCT4, KLF4, c-Myc), and epithelial–mesenchymal transition (ZEB1/2, SNAIL, Vimentin) [20,22]. Downregulation of miR-145 arises from a multifaceted regulatory network: (1) lncRNA sponging by MALAT1 and HOTAIR, which sequester miR-145 in NSCLC cells; (2) promoter hypermethylation at the miR-145 host gene locus (5q32), particularly in TP53-mutant tumors; and (3) transcriptional repression by oncogenic signals such as EGFR-activated ERK, which suppresses miR-145 biogenesis [21,23,24]. Clinically, reduced miR-145 correlates with advanced TNM stages, increased metastatic burden, and enrichment of cancer stem cell (CSC) populations [25]. Critically, miR-145 antagonizes CSC properties by disrupting ADAM17-mediated Notch signaling and BMI1-driven self-renewal pathways, explaining its potent metastasis-suppressing activity [20]. Therapeutic restoration of miR-145 via nanoparticle delivery has shown promise in preclinical models, reducing invasion by >60% and resensitizing tumors to EGFR inhibitors, positioning it as a compelling target for RNA-based therapies in NSCLC.

Complementing our ceRNA network findings, clinical database analysis revealed significant co-expression of FN1 and CCND1 across NSCLC tumors, supporting their functional linkage in disease pathogenesis. Importantly, elevated FN1 expression demonstrated clear association with adverse patient outcomes, independently validating its role as a clinically relevant prognostic biomarker in lung adenocarcinoma.

The broad age range of patients in the GSE135304 cohort (26–93 years, mean 65.3 years), as reported in Section 3.1, aligns with the epidemiological profile of NSCLC, where incidence spans young adults to the elderly, with a median diagnosis age of approximately 70 years, but including rare cases in younger (<40 years, ~1–5% of cases) and older (≥85 years, ~14% of cases) populations [26,27,28]. This heterogeneity arises from diverse risk factors, including cumulative tobacco exposure, environmental carcinogens (e.g., radon, pollution), and genetic predispositions (e.g., EGFR or ALK mutations being more common in younger patients) [29,30]. While age can influence NSCLC’s molecular landscape, for example, younger patients show higher rates of EGFR mutations (~40% in <40-year-olds vs. ~15% in >70-year-olds) [31,32], the miR-145-mediated ceRNA network and its hub genes (FN1, CCND1) appear robust across ages. Studies indicate that miR-145-3p downregulation in NSCLC tissues and plasma is consistent regardless of patient age [33] and that FN1/CCND1 expression shows no significant age-related variation in NSCLC cohorts [34]. To address potential confounding by age, we conducted a sensitivity analysis using the GSE135304 dataset, stratifying differential expression of miR-145-3p, FN1, and CCND1 by age subgroups (<65 vs. ≥65 years). This analysis confirmed consistent downregulation of miR-145-3p (*p* < 0.05) and upregulation of FN1/CCND1 (*p* < 0.05) in both subgroups, with no significant age-expression interaction (*p* > 0.1, ANOVA). These findings suggest that the LOC729919/LOC100134412–miR-145–FN1/CCND1 axis operates independently of age, ensuring the reliability of our molecular insights. The broad age range thus enhances the external validity of our study, capturing the full spectrum of NSCLC biology without compromising the consistency of the ceRNA network findings.

FN1, the terminal effector in this network, encodes an ECM glycoprotein with well-documented roles in promoting metastasis through integrin-mediated signaling [35,36]. FN1 drives NSCLC metastasis through integrin-mediated FAK/SRC signaling while conferring cisplatin resistance via Wnt/β-catenin activation through direct integrin-β1 binding, in addition, silencing of FN1 sensitizes resistant cells by reducing IC50 values and restoring apoptosis [37]. FN1’s pro-metastatic activity is amplified by exosomal LRG1, which forms a physical complex with FN1 to trigger EMT transcription factors and accelerate distant colonization [38]. Non-coding RNAs reciprocally regulate FN1: tumor-suppressive hsa_circ_0050386 binds SRSF3 to block FN1 pre-mRNA splicing and suppress metastasis [39], while oncogenic Lnc-PDZD7-3 upregulates FN1 to enhance MMP2/9-mediated invasion [40]. This positions FN1 as a nexus for ECM reprogramming and therapeutic resistance in NSCLC.

CCND1 (cyclin D1) serves as a critical cell cycle regulator in NSCLC, where its overexpression drives tumor proliferation by accelerating G1/S transition and is associated with poorly differentiated histology and reduced local relapse rates [34]. This oncogene is directly targeted and suppressed by multiple tumor-suppressive miRNAs, including miR-134 and miR-146a-5p, which bind its 3’UTR to inhibit expression. Indeed, low levels of these miRNAs in NSCLC correlate with CCND1 upregulation, promoting uncontrolled cell growth and metastasis [41,42]. The CCND1 A870G polymorphism further modulates NSCLC risk, where the GG genotype intensifies smoking-associated carcinogenesis (≥40 pack-years) and improves response to platinum-based chemotherapy, suggesting its utility in treatment stratification [43]. In our ceRNA network, CCND1 is derepressed through lncRNA-mediated sequestration of miR-145, amplifying its oncogenic activity. This regulatory disruption represents a convergence point for cell cycle dysregulation and therapeutic vulnerability in NSCLC.

Pathway enrichment analysis of differentially expressed genes highlighted key processes driving malignant progression, including TGF-β signaling, DNA damage response (ATM-dependent), Hippo signaling regulation, and cell cycle dysregulation. These pathways intersect at critical nodes of cancer biology: TGF-β promotes EMT and immunosuppression [44,45]; ATM coordinates genomic stability [46]; and Hippo signaling modulates organ size and tumor growth [47,48]. The convergence of these pathways in our dataset suggests coordinated reprogramming of cellular homeostasis in malignant nodules, with FN1 and CCND1 serving as a physical bridge between TGF-β-mediated EMT and integrin-driven survival signaling.

The pathway enrichment analysis identified several dysregulated pathways critical to NSCLC progression, including the ATM-dependent DNA damage response, TGF-β signaling, cell cycle regulation, Hippo signaling, and TGF-β-mediated EMT. These pathways are interconnected and collectively drive malignant transformation, with robust evidence from preclinical and clinical studies supporting their contributions to tumor initiation, proliferation, invasion, metastasis, and therapeutic resistance. ATM encodes ataxia-telangiectasia mutated (ATM) a cell cycle checkpoint kinase involved in cellular response to double stranded DNA breaks that belongs to the PI3/PI4-kinase family. The ATM-dependent DNA damage response pathway is frequently altered in NSCLC, with ATM mutations reported in approximately 9% of lung adenocarcinomas (LUAD) and 4% of squamous cell carcinomas, leading to genomic instability and enhanced tumor survival under genotoxic stress [49,50,51]. ATM activation facilitates DNA double-strand break repair and cell cycle arrest via checkpoint kinases; its dysregulation promotes unchecked proliferation and resistance to chemotherapy and radiotherapy by impairing homologous recombination repair [52]. Recent studies demonstrate that ATM-mediated translocation of RanBPM stabilizes DNA damage response proteins, enhancing NSCLC invasion in vivo models [53].

TGF-β signaling plays a dual role in NSCLC acting as a tumor suppressor in early disease stages by inhibiting proliferation but being pro-metastatic in advanced disease by inducing EMT and immune evasion [54,55]. Both canonical (Smad-dependent) and non-canonical (e.g., MAPK/PI3K) TGF-β pathways remodel the ECM, promote angiogenesis, and facilitate metastatic dissemination, with elevated TGF-β levels correlating with poor prognosis in NSCLC cohorts [56]. Dysregulation of inhibitory Smads (e.g., Smad7) further enhances TGF-β-driven fibrosis and tumor-stromal interactions, accelerating disease progression [57].

Cell cycle dysregulation is a hallmark of NSCLC, with aberrations in regulators such as cyclins (e.g., CCND1) and cyclin-dependent kinases (CDKs) driving uncontrolled G1/S phase transition and genomic instability [58,59,60]. Concomitant defects in multiple cell cycle checkpoints, including p53 and Rb pathways, exacerbate adverse outcomes, with deregulated cell cycle genes serving as independent prognostic markers and predictors of chemotherapy response in NSCLC [61]. Single-cell analyses further reveal that cell cycle-enriched gene signatures in LUAD subtypes promote stemness and metastatic potential [58].

The Hippo signaling pathway, through its core kinases (MST1/2, LATS1/2) and effectors (YAP/TAZ), regulates organ size and suppresses tumorigenesis. Its inactivation in NSCLC leads to YAP/TAZ nuclear translocation, fostering proliferation, EMT, and chemoresistance. Dysregulated Hippo components, such as AMOT, interact with hypoxia-inducible factors to drive metastasis, with YAP overexpression associated with advanced TNM stages and reduced survival [62,63]. Crosstalk with TGF-β and Wnt pathways amplifies Hippo’s role in NSCLC invasion [64].

TGF-β-mediated EMT is a pivotal mechanism for NSCLC metastasis, where TGF-β induces loss of E-cadherin and upregulation of vimentin/N-cadherin, enabling invasion and cancer stem cell-like properties [54]. This transition reprograms amino acid metabolism to sustain EMT, correlating with lymph node metastasis and poor overall survival in NSCLC cohorts [65]. Gene signatures of TGF-β-driven EMT predict metastasis-free survival, underscoring its potential as a therapeutic target [66,67]. These pathways converge on shared effectors like FN1 and CCND1, as identified in our ceRNA network, reinforcing their relevance to the miR-145 axis and highlighting opportunities for combinatorial therapeutic strategies in NSCLC.

Several limitations warrant consideration. First, the initial transcriptomic datasets (GSE135304: blood RNA; GSE203510: plasma miRNAs) were derived from peripheral circulation rather than tumor tissue, potentially limiting direct interpretation of cancer cell-specific regulatory dynamics. Future validation in tissue-derived NSCLC cohorts is essential to confirm spatial relevance within the tumor microenvironment. Second, insufficient metadata (e.g., detailed smoking history, treatment status, or molecular subtypes) restricted our ability to assess confounding effects on miRNA/mRNA expression patterns. Third, while statistically significant, the observed FN1-CCND1 co-expression correlation (r = 0.13) was relatively weak, suggesting additional regulatory layers beyond miR-145 sponging may influence their relationship. Orthogonal validation using cancer tissue-specific datasets and mechanistic studies dissecting direct protein interactions are warranted to establish functional nexus. Fourth, functional characterization of LOC729919 remains sparse, necessitating studies to delineate its binding partners and downstream effects. Fifth, the limited paired tumor and normal lung tissue samples from NSCLC patients, potentially failing to capture the full spectrum of molecular heterogeneity in NSCLC. Finally, the precise hierarchy within the ceRNA network, particularly how lncRNA stoichiometry influences miR-145 availability, needs experimental clarification.

## 5. Conclusions

In conclusion, our study establishes a novel lncRNA-mediated ceRNA axis (LOC729919/LOC100134412–miR-145–FN1/CCND1) as a driver of NSCLC pathogenesis. Experimental validation confirmed consistent miR-145-3p downregulation across cellular and clinical NSCLC models, reinforcing its role as a master tumor suppressor. While FN1 elevation demonstrates clear clinical relevance through its association with adverse outcomes, CCND1’s lack of direct survival linkage suggests its primary role may lie in cell cycle dysregulation rather than metastatic progression. Targeting this axis, particularly FN1-mediated pathways, represents a promising therapeutic strategy against advanced NSCLC.

## Figures and Tables

**Table 1:** Top enriched pathways for NSCLC DEGs.

Term	Genes	Adjusted *p*-Value
DNA damage response (only ATM dependent) WP710	CCND2; SMAD3; CCND1; SHC1; SOD2; MAP3K7	5.55 × 10^−5^
TGF-beta Signaling Pathway WP366	SMAD3; COL1A2; CCND1; SHC1; FN1; MAP3K7	1.52 × 10^−4^
Hypothesized Pathways in Pathogenesis of Cardiovascular Disease WP3668	SMAD3; SHC1; FBN1	4.51 × 10^−4^
Cell cycle WP179	CCND2; CDC23; SMAD3; CCND1; BUB1	8.17 × 10^−4^
The influence of laminopathies on Wnt signaling WP4844	CCND1; AGO2; HMGA2	0.001227
Mammary gland development pathway—Puberty (Stage 2 of 4) WP2814	CCND1; FN1	0.002711
Pathways Regulating Hippo Signaling WP4540	NTRK2; SMAD3; PRKAG1; CDH18	0.002972
The Overlap Between Signal Transduction Pathways that Contribute to a Range of LMNA Laminopathies WP4879	SMAD3; AGO2; HMGA2	0.004507
Canonical and non-canonical TGF-B signaling WP3874	SMAD3; LOX	0.004652
TGF-B Signaling in Thyroid Cells for Epithelial–Mesenchymal Transition WP3859	SMAD3; FN1	0.005213

**Table 2:** Top 10 enriched pathways of miR-145-3p targets for NSCLC.

Terms	Genes	Adjusted *p*-Value	GO
**Biological Process**			
Positive Regulation of Mitotic Cell Cycle Phase Transition	CDC23; CCND2; CCND1	0.013	1901992
Regulation of Purine Nucleotide Catabolic Process	PRKAG1; OGT	0.013	0033121
Regulation of Transforming Growth Factor Beta Production	SMAD3; FN1	0.013	0071634
Regulation of Carbohydrate Catabolic Process	PRKAG1; OGT	0.013	0043470
Regulation of ATP Metabolic Process	PRKAG1; OGT	0.014	1903578
Regulation of Generation of Precursor Metabolites and Energy	PRKAG1; OGT	0.021	0043467
Cell–Matrix Adhesion	VCAM1; FN1; ANGPTL3	0.021	0007160
Mitotic Cell Cycle Phase Transition	CDC23; CCND2; CCND1	0.022	0044772
Negative Regulation of Fat Cell Differentiation	SMAD3; ZFP36L2	0.032	0045599
Positive Regulation of G1/S Transition of Mitotic Cell Cycle	CCND2; CCND1	0.032	1900087
**Cellular Component**			
Cyclin-Dependent Protein Kinase Holoenzyme Complex	CCND2; CCND1; CDK5R1	0.001	0000307
Serine/Threonine Protein Kinase Complex	CCND2; CCND1; CDK5R1	0.003	1902554
Platelet Alpha Granule Lumen	SERPINA1; FN1	0.063	0031093
Platelet Alpha Granule	SERPINA1; FN1	0.065	0031091
Endoplasmic Reticulum Lumen	SERPINA1; FN1; CKAP4	0.065	0005788
Intracellular Membrane-Bounded Organelle	SERPINA1; SMAD3; ZNF281; PRKAG1; ESRRG; ZC3H6; ZFP36L2; PURB; RAB11FIP1; CCND2; CCND1; BUB1; OGT; CDK5R1	0.065	0043231
NSL Complex	OGT	0.094	0044545
Collagen-Containing Extracellular Matrix	SERPINA1; FN1; ANGPTL3	0.094	0062023
Lamellar Body	CKAP4	0.094	0042599
Nucleus	PURB; CCND2; SMAD3; CCND1; ZNF281; PRKAG1; ESRRG; ZC3H6; BUB1; OGT; ZFP36L2; CDK5R1	0.094	0005634
**Molecular Function**			
Cyclin-Dependent Protein Serine/Threonine Kinase Activator Activity	CCND2; CCND1; CDK5R1	2.22	0061575
Protein Kinase Regulator Activity	CCND2; CCND1; PRKAG1; CDK5R1	6.52	0019887
Cyclin-Dependent Protein Serine/Threonine Kinase Regulator Activity	CCND2; CCND1; CDK5R1	7.93	0016538
Protein Serine/Threonine Kinase Activator Activity	CCND2; CCND1; CDK5R1	0.002	0043539
Kinase Binding	CCND2; SMAD3; CCND1; PRKAG1; CDK5R1	0.005	0019900
Protein Kinase Activator Activity	CCND2; CCND1; CDK5R1	0.005	0030295
Protein Kinase Binding	CCND2; SMAD3; CCND1; PRKAG1; CDK5R1	0.007	0019901
Kinase Activity	GK5; CDK5R1	0.057	0016301
cAMP-dependent Protein Kinase Activity	PRKAG1	0.057	0004691
DEAD/H-box RNA Helicase Binding	SMAD3	0.057	0017151

## Data Availability

The original contributions presented in this study are included in the article. Further inquiries can be directed to the corresponding authors.
